# Clinical Impact of MRI-Guided Intracavitary–Interstitial Brachytherapy in the Curative Management of Advanced-Stage Cervical Cancer

**DOI:** 10.3390/curroncol32030136

**Published:** 2025-02-26

**Authors:** Antje Wark, Laura Hüfner, Eva Meixner, Jan Oelmann, Laila König, Simon Höne, Katja Lindel, Jürgen Debus, Nathalie Arians

**Affiliations:** 1 Department of Radiation Oncology, Heidelberg University Hospital, Im Neuenheimer Feld 400, 69120 Heidelberg, Germany; antje.wark@klinikum-karlsruhe.de (A.W.);; 2 Department of Radiation Oncology, Städtisches Klinikum Karlsruhe, 76133 Karlsruhe, Germany; 3 Heidelberg Institute of Radiation Oncology (HIRO), 69120 Heidelberg, Germany; 4 National Center for Tumor Diseases (NCT), 69120 Heidelberg, Germany; 5 Department of Radiation Oncology, Göttingen University Hospital, 37075 Göttingen, Germany; 6 Department of Radiation Oncology, Krankenhaus Barmherzige Brüder Regensburg, 93049 Regensburg, Germany; 7 German Cancer Consortium (DKTK), 69120 Heidelberg, Germany; 8 Heidelberg Ion-Beam Therapy Center (HIT), Heidelberg University Hospital, 69120 Heidelberg, Germany; 9 Clinical Cooperation Unit Radiation Oncology, German Cancer Research Center (DKFZ), 69120 Heidelberg, Germany

**Keywords:** advanced cervical cancer, image-guided adaptive brachytherapy, MRI-guidance

## Abstract

This study investigates the clinical efficacy of MRI-based adaptive brachytherapy (IGABT) using combined intracavitary and interstitial techniques in the curative treatment of patients with advanced cervical cancer (LACC). A retrospective analysis was conducted on 149 LACC patients treated at a single center. The therapeutic protocol included intensity-modulated external beam radiotherapy (IMRT) and IGABT. Dosimetric parameters were evaluated for relevance for local control (LC), progression-free survival (PFS), and overall survival (OS) using Kaplan–Meier estimation, Cox regression, and log-rank test. Patients predominantly presented with stage III/IV tumors (81%, FIGO 2018). The median high-risk clinical target volume (hrCTV) was 34 cm^3^, with a median D90% dose of 88.9 GyEQD2. At 24 months, OS, PFS, and LC rates were 86%, 57%, and 81%, respectively. FIGO stage, tumor volume, and histology were significant predictors of PFS. Higher total hrCTV doses were strongly correlated with improved LC and PFS, emphasizing the importance of precise dosimetric optimization in IGABT and confirming the critical role of IGABT in achieving very good LC rates for LACC. The reported LC rates are comparable to landmark studies, such as INTERLACE and KEYNOTE-A18. This study validates the effectiveness of MRI-guided IGABT in enhancing local tumor control in advanced-stage cervical cancer while providing insights into the prognostic implications of dosimetric parameters such as hrCTV and point A. Future research should address the persistent challenge of distant metastases by exploring the integration of novel systemic treatment options.

## 1. Introduction

Cervical cancer remains to this day a significant public health challenge worldwide, despite advances in prevention and early detection, such as the widespread adoption of HPV vaccination and screening with Pap smear tests [1]. Locally advanced cervical cancer (LACC) continues to present a substantial burden even in high-income countries, due to its aggressive nature and the complexities involved in its treatment.

The management of LACC typically consists of a multimodal approach, with a combination of external beam radiotherapy (EBRT) and brachytherapy as well as radiosensitising chemotherapy. While alternative percutaneous boost techniques have been explored, brachytherapy remains a cornerstone of definitive radiotherapy for cervical cancer, delivering high-dose radiation directly to the tumor while minimizing exposure to surrounding healthy tissues.

Recent advancements in brachytherapy techniques have significantly enhanced the therapeutic ratio for patients with LACC. MRI-based image-guided adaptive brachytherapy (IGABT) allows a more precise target volume definition. The combination of this technique with intracavitary and interstitial brachytherapy offers technical advantages by enhanced target coverage including involved parametrial tissue while sparing organs at risk, thus mitigating treatment-related toxicity.

Despite the availability of treatment guidelines published by the European Society of Gynecological Oncology (ESGO) and the European Society of Radiotherapy and Oncology (ESTRO) [2], treatment delivery continues to vary significantly across regions and between clinical institutions.

This paper examines the clinical outcomes, technical aspects, and future prospects of modern MRI-based interstitial IGABT, emphasizing its potential to enhance survival rates and reduce treatment-related toxicities in patients with LACC. We evaluated dosimetric parameters critical for oncological outcomes. Key endpoints assessed include overall survival (OS), progression-free survival (PFS), and local control (LC).

## 2. Materials and Methods

For this retrospective analysis, we reviewed all patients with LACC, who were treated by definitive radio(-chemo)therapy including MRI-guided brachytherapy (BRTH) in a curative setting at the Department of Radiation Oncology of the Heidelberg University Hospital between July 2015 and April 2022. A total of 580 brachytherapy plans from 149 patients were available for the analysis of the radiation parameters of brachytherapy. Patients were classified according to the International Federation of Gynaecology and Obstetrics (FIGO) 2018 staging criteria [3]. Included were patients with localized or oligometastatic disease (FIGO stages IB, non-suitable for the surgical approach, to IVB). Inclusion criteria were the use of a tandem-ovoid-hybrid brachytherapy applicator and MRI-based brachytherapy planning. Exclusion criteria were a palliative treatment indication or previous hysterectomy. Simultaneous chemotherapy was not a requirement.

Each patient indication for definitive radio(chemo)therapy was confirmed by a tumor board before treatment initiation.

Target volume definition of EBRT, dose constraints to organs at risk, and plan optimization were performed in accordance with the RTOG consensus guidelines [4] while adhering to the ALARA (as low as reasonably achievable) principle. For external beam radiotherapy, a single fraction dose of 1.8 Gy to a total dose of 45 to 50.4 Gy was prescribed and administered using a VMAT technique (volumetric arc therapy) with 6 MV photons. Dose escalation to macroscopic lymph node metastases, mostly using a simultaneous integrated boost concept (SIB), was applied if appropriate. In total, 22 patients received percutaneous radiochemotherapy (CRT) at external clinics and were referred to the study center for the brachytherapy boost irradiation. For 126 patients, detailed EBRT treatment plans were available for assessment.

After administration of a total dose of at least 36 Gy, tumor response was assessed by diagnostic MRI. Based on this imaging as well as tumor spread on initial MRI brachytherapy applicator implantation was planned. Brachytherapy was conducted as high-dose-rate (HDR) brachytherapy using an Iridium-192 afterloader with tandem hybrid applicators from Elekta AB, Sweden, with the “Utrecht” applicator being used in 75% of patients and the “Geneva” applicator in 25% of cases. At least at one brachytherapy session, an MRI was performed in addition to a planning CT after insertion of the applicator, preferably at the first fraction if possible. The applicator insertions per patient were performed once a week while still under external beam radiotherapy or a maximum of two times a week thereafter with only one administration per insertion. The brachytherapy boost was prescribed as 4 fractions of 7 Gy single dose to the high-risk clinical target volume (hrCTV) which was defined based on MRI according to the Groupe Européen de Curiethérapie and the European Society for Radiotherapy and Oncology (GEC-ESTRO) guidelines [5,6] and included the cervix as well as any residual tumor spread. Constraints for the organs at risk (OAR) rectum, bladder, sigmoid colon, and bowel were given according to the GEC-ESTRO recommendations.

For the retrospective dosimetric analysis, the dose coverage and volumes of the hrCTV, the dose exposure of the OARs bladder, rectum, and bowel (D0.1cc, D1cc, D2cc) as well as the median dose at point A were determined. In addition, the dose prescribed to the Dmean of the PTV (planning target volume), and the organ exposure doses were taken from the EBRT treatment plans. Equivalent doses in 2 Gy fractions (EQD2) from EBRT and BRTH were calculated using an α/β of 10 Gy for the tumor and α/β of 3 Gy for the OARs. Cumulative doses were calculated as a summation of the mean dose from EBRT and D2cc from each brachytherapy fraction for organs at risk (OAR), respectively, the D90% for hrCTV.

Follow-up examinations consisted of clinical examination and pelvic MRI every three months in the first year, every six months for the next two years, and annually thereafter. Restaging, including CT thorax and upper abdomen, was usually repeated annually or at suspicion of locoregional progression. Vital status was evaluated via the national citizen registry.

Local control (LC) and progression-free survival (PFS) were defined as the time from the last brachytherapy treatment to the first date of recurrence (for PFS local, regional, or distant recurrence) or the date of last follow-up. Death was censored. Overall survival was equally defined as the time from the last brachytherapy treatment to the date of death of any causes.

Treatment-related toxicity was evaluated retrospectively from the institutional database and clinical follow-up assessments and documented according to the Common Terminology Criteria for Adverse Events (CTCAE) classification (v5). Acute toxicity was defined as adverse events occurring during and up to 6 weeks after radio(chemo)therapy, chronical toxicity was defined as symptoms persisting more than 3 months after completion of therapy.

Statistical analysis was conducted using SPSSv28 (IBM Corp., Cohortonk, New York, NY, USA). The dosimetric evaluation included the historic point “A” and the hrCTV. A correlation of these parameters with progression-free survival (PFS) and local control (LC) was investigated using Cox proportional hazard regression. Univariate analysis was performed using the log-rank test. Survival statistics were calculated using the Kaplan–Meier estimator. Pearson’s test and Spearman’s rho test were employed to evaluate associations between usage of interstitial needles and hrCTV volume and dose, as well as point A.

Ethics approval for the study was granted by the Heidelberg University ethics committee (#S-808/2019).

## 3. Results

Between July 2015 and April 2022, a total of 149 patients were treated for LACC at Heidelberg University Hospital by curatively intended definitive radio(chemo)therapy including IGABT. An overview of patient characteristics is listed in Table 1.

The average age at diagnosis was 55 years, with the youngest patient being 31 years old, and the oldest 88 years old. Histologically, 124 patients (83%) were diagnosed with squamous cell carcinoma, 23 patients (15%) with adenocarcinoma, and 2 patients (1%) with neuroendocrine differentiated carcinoma.

Prior to radiation therapy, various diagnostic methods, including MRI, cystoscopy, and rectoscopy, if necessary, were employed to rule out potential infiltration of surrounding organs.

The most common tumor stage was cT2b in 68 patients (46%). There were 20 cases of cT3 (13%) and 22 cases (15%) of cT4 stage; among these patients, there were 18 cases (12%) with suspicion of urinary bladder infiltration and 6 cases (4%) of rectal infiltration. Vaginal infiltration was present in 78 patients (52%). Actually, the histological proof of organ infiltration by biopsy was only performed in four patients. The others were diagnosed as cT4 by cystoscopy or rectoscopy (n = 8) or MRI only (n = 10).

At initial diagnosis, 106 patients (71%) had pelvic lymph node involvement and 39 patients (26%) had para-aortic lymph node metastases determined by lymphadenectomy or imaging. As part of the staging process, 94 patients had pelvic and/or paraaortal lymphonodectomy, 79 patients (53%) underwent pelvic lymphadenectomy, and 75 patients (50%) underwent paraaortic lymphadenectomy. In 69 patients, lymph node metastases were found. It is noteworthy that 33 of them still had nodal metastasis in situ at the time of radiotherapy and received nodal boosting. In total, 37 patients were diagnosed as N+ pelvic or pelvic and paraaortal by imaging (nodal size > 1 cm on MRI or CT and diffusion restriction on MRI).

In total, 42 patients (28%) presented with distant metastases at initial diagnosis. Among these, 39 patients (26%) had para-aortic lymph node metastases, classified as M1a stage, while 6 patients (4%) were oligometastatic. One with osseus metastasis, one with liver metastasis, one with liver metastasis and thoracal as well as inguinal lymphatic metastases, and three with thoracal lymphatic metastases. Metastases were treated with additional radiotherapy, surgery, and/or chemotherapy. It is noteworthy that none of them had locoreginal recurrence. Three of them showed distant recurrence and two died during follow-up.

**Figure 1:**
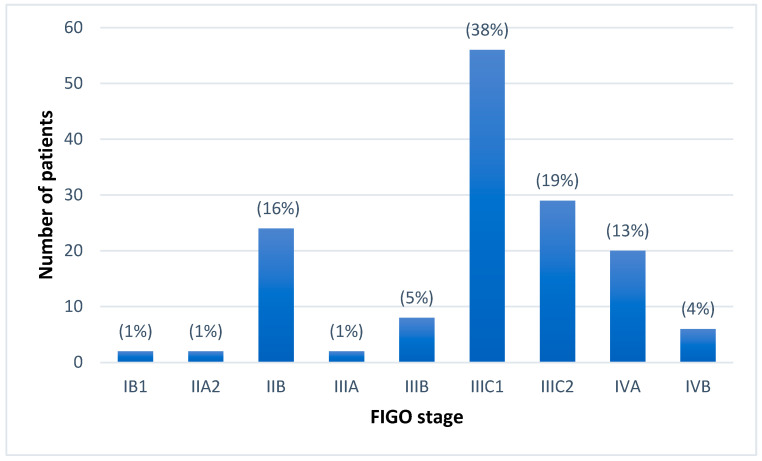
Distribution of staging according to the FIGO 2018 classification for cervical cancer patients (n = 149). The bars represent the absolute number of patients and the corresponding percentage for each stage, highlighting the prevalence of each stage within the cohort.

Overall, patients were mostly in advanced stages: 81% of the patients were staged as FIGO stage III or IV. The distribution of FIGO stages in the collective is depicted in Figure 1.

The median time from the date of initial diagnosis until the start of radiotherapy was 53 days (3–871 days) for the entire cohort, with the longest interval in a patient who did initially refuse treatment. Patients who did not undergo LNE had a shorter interval from initial diagnosis to treatment initiation with a median of 39 days (3–510 days), whereas patients who received LNE had a median interval of 60 days (10–871 days).

The median overall treatment time (OTT) was 51 days, with one patient completing treatment in 41 days, while the longest duration was 105 days due to repeated acute infections and severe leukopenia, which delayed chemotherapy.

Percutaneous irradiation to the pelvic +/− paraaortal region was applied with a mean dose of 44.25 Gy EQD2 (43.9–54.25 Gy EQD2). In 41 patients (28%), EBRT included the paraaortal region. In total, 43 patients (29%) received boost irradiation to pelvic or paraaortal lymph node metastases that remained in situ at the start of treatment. In total, 38 patients received the boost as a simultaneous integrated boost. In 25 cases, it was prescribed in 2.2 Gy single doses to a cumulative dose of 55 Gy; in 7 patients, it was to a cumulative dose of 56 Gy in 2 Gy single doses; in 4 patients, it was to a cumulative dose of 57.5 Gy in 2.3 Gy single doses; in 1 patient, it was to 58.8 Gy in 2.1 Gy single doses; and in 1 patient, it was to 60 Gy in 2.4 Gy single doses. In total, 5 patients received the boost sequentially with a total dose of 5.4 Gy, respectively, 9 Gy in 1.8 Gy single doses in two cases each and 12.5 Gy in 2. 5 Gy single doses in one case.

Concurrent chemotherapy was administered to 138 patients (93%). Of these, 124 patients (90%) received cisplatin weekly, 8 patients (5%) were treated with carboplatin, and 3 patients (2%) were switched from cisplatin to carboplatin during treatment due to the onset of hearing loss in two cases and an allergic reaction with exanthema in one case. An average of 5 courses was administered. In three patients (2%), concurrent chemotherapy regime consisted of mitomycin and 5-FU.

In total, 20 patients (13%) were treated with intracavitary (IC) IGABT alone, and 129 patients (88%) with combined intracavitary and interstitial (IS) IGABT. In the 129 patients treated with IC/IS IGABT, at least two needles were used per fraction in 103 patients (80%). The median number of interstitial needles per fraction was three (range 0 to 9).

The median hrCTV volume was 34 cm^3^ (range 7–158 cm^3^) and the median D90% of the hrCTV was 88.9 Gy EQD2 (range 77.9–111.8 Gy EQD2). The average dose at point A was 8.25 Gy (range 2.84–29.61 Gy). Detailed dose–volume parameters for brachytherapy as well as cumulative doses from brachytherapy and EBRT are depicted in Table 2.

The average follow-up period was 22 months (range 0.4 to 80.7 months). The loss to follow-up was mostly due to patients receiving initial treatment and further oncological care at other institutions.

**Figure 2:**
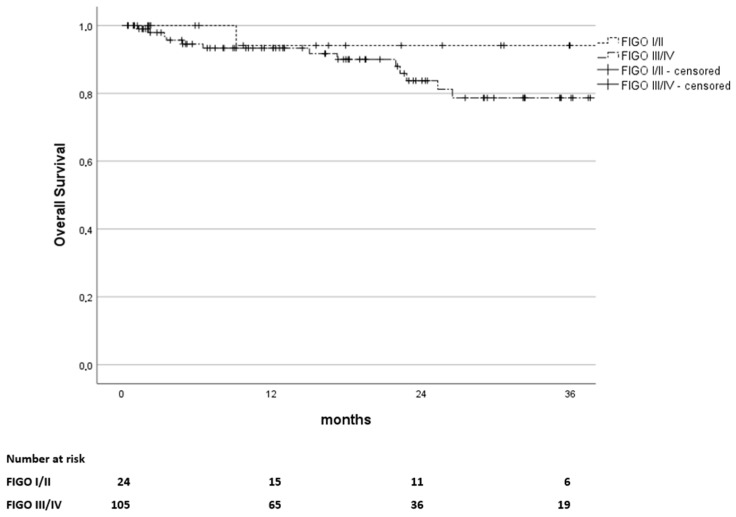
Kaplan–Meier estimates of overall survival in months since the end of treatment, stratified by FIGO stages I and II versus stages III and IV (*p* = 0.202). The number of patients at risk is stated below.

The overall survival (OS) rates were 94%, 86%, and 82% at 12, 24, and 36 months. The Kaplan–Meier curves for OS stratified by FIGO stage I and II vs. III and IV are depicted in Figure 2.

In the follow-up period, local recurrence was diagnosed in 18 patients (12%), resulting in an actuarial local recurrence-free survival rate of 90% at one year, 81% at two years, and 76% at three years.

**Figure 3:**
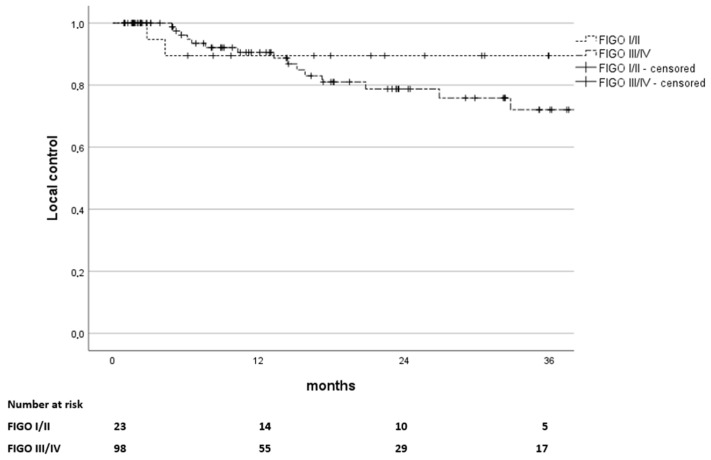
Kaplan–Meier estimates of local-control rate in months since the end of treatment, stratified by FIGO stages I and II versus stages III and IV (*p* = 0.311). The number of patients at risk is stated below.

Local control (LC) was 90% at 12, 24, and 36 years for early stages as opposed to 90%, 79%, and 72% for advanced stages, as depicted in Figure 3.

**Figure 4:**
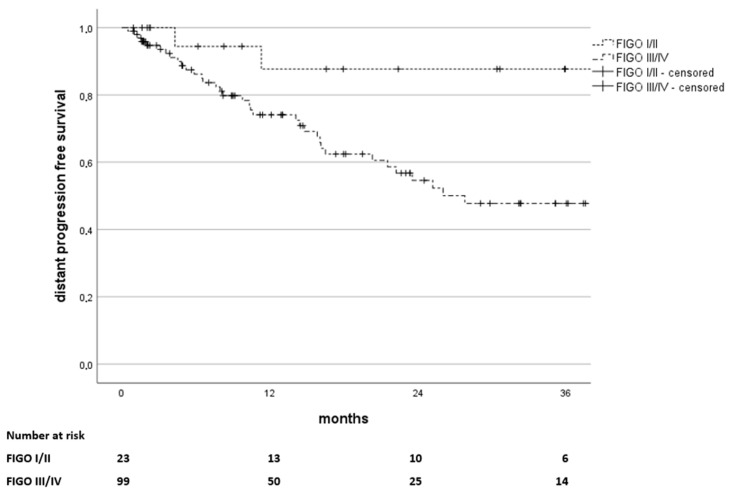
Kaplan–Meier estimates of distant progression-free survival in months since the end of treatment, stratified by FIGO stages I and II versus stages III and IV (*p* = 0.027). The number of patients at risk is stated below.

Pelvic nodal progression occurred in nine cases (6%) with Nodal progression-free survival (NPFS) rates of 95%, 89%, and 87% at 12, 24, and 36 months. Distant metastases were diagnosed in 40 patients (27%) with distant progression-free survival (DPFS) rates of 77%, 61%, and 56% at 12, 24 and 36 months. A significant difference between DPFS rates was observed between FIGO stages I/II and III/IV with DPFS rates of 88% at 12, 24, and 36 months for FIGO stages I and II as opposed to 74%, 55%, and 48% for FIGO stages III and IV (*p* = 0.027) as depicted in Figure 4. Among the distant recurrences, we observed a total of 11 paraaortal recurrences, 2 of them were isolated paraaortal recurrences, and 6 of these patients already presented with paraaortal lymphnode metastasis at the time of diagnosis (M1a).

**Figure 5:**
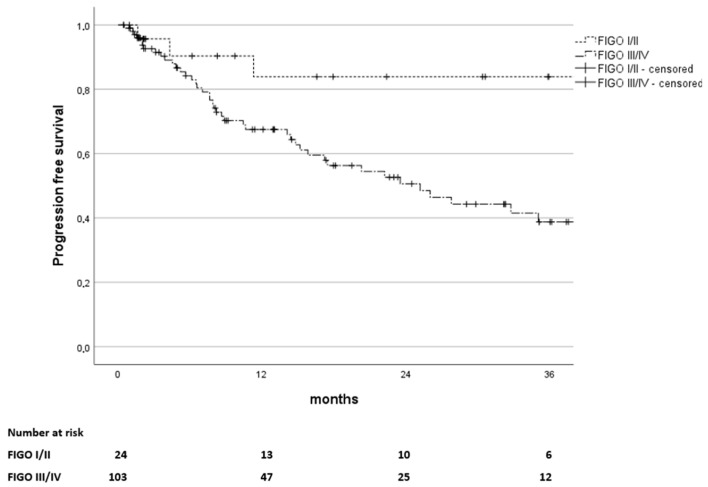
Kaplan–Meier estimates of progression-free survival in months since the end of treatment, stratified by FIGO stages I and II versus stages III and IV (*p* = 0.023). The number of patients at risk is stated below.

The overall progression-free survival rate (PFS) was 70% at one year, 57% at two years, and 48% at three years with a significant difference between FIGO stages I/II and stages III/IV (*p* = 0.023), depicted in Figure 5. PFS rates were 84% at 12, 24, and 36 months for early stages as opposed to 68%, 51%, and 39%, respectively, for advanced stages.

**Figure 6:**
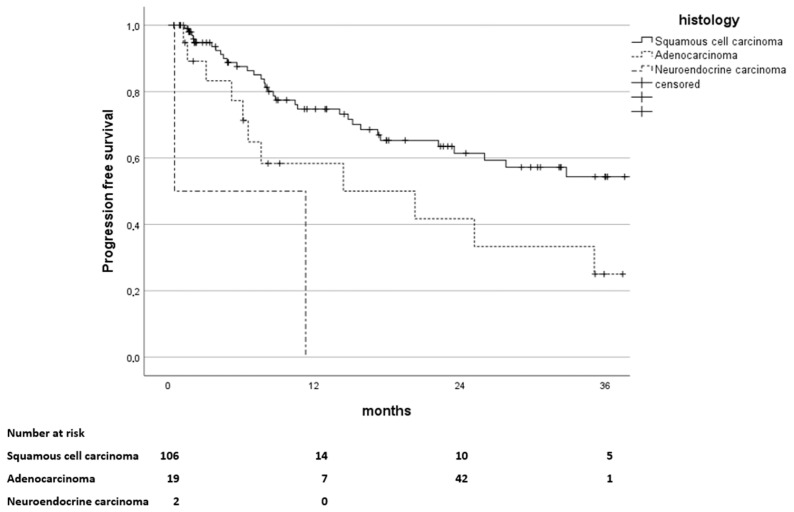
Kaplan–Meier estimates of progression-free survival in months since the end of treatment, stratified by histology: Squamous cell carcinoma, Adenocarcinoma, Neuroendocrine carcinoma (*p* = 0.002). The number of patients at risk is stated below.

Stratification for histology showed no significant differences in OS rates for squamous cell carcinoma (SCC) as opposed to adenocarcinoma (*p* = 0.895). However, there was a significant difference in PFS rates due to significantly more events in patients with adenocarcinoma histology (*p* = 0.002). PFS in the SCC subgroup was 78%, 61%, and 54% at 12, 24, and 36 months opposed to 58% at 12 months and 42% at 24 months in the Adenocarcinoma cohort. Corresponding Kaplan-Maier curves are graphically depicted in Figure 6.

A Cox regression analysis was conducted to evaluate the impact of cumulative EQD2 from brachytherapy and EBRT to the hrCTV, hrCTV volume, histological type (Adenocarcinoma or Squamous cell carcinoma), and use of chemotherapy on LC. The results of the analysis are summarized in Table 3.

The analysis showed that a higher total dose of the hrCTV was significantly associated with improved LC (*p* = 0.003). The negative coefficient suggests that as the total dose increases, the risk of local recurrence decreases. An increase in the hrCTV volume, indicating residual tumor spread protruding beyond the cervix, was significantly associated with an increased risk of local recurrence (*p* = 0.005). The histological type did not show a significant association with LC (*p* = 0.777). Regarding the effect of chemotherapy on LC, no statistical significance was reached in this model (*p* = 0.122), although the negative coefficient indicates a trend towards improved LC with chemotherapy use.

Another Cox regression analysis was conducted to investigate the impact of several variables on progression-free survival (PFS). The variables included in this statistical model were total dose from brachytherapy, hrCTV volume, use of chemotherapy, histological type (adenocarcinoma vs. squamous cell carcinoma), nodal stage, and metastasis stage. The results of the analysis are summarized in Table 4. The analysis indicated that an increased total dose of the hrCTV, resulting from a higher dose from brachytherapy, was significantly associated with improved PFS (*p* = 0.013). The negative coefficient suggests that as the dose increases, the risk of progression decreases. A larger hrCTV volume was significantly associated with worse PFS (*p* = 0.020), suggesting that as the hrCTV volume increases, the risk of progression also increases. The variable “chemotherapy” was most likely not significantly associated with PFS improvement in this model due to more than 90% of patients receiving concurrent chemotherapy. The histology type had a significant impact on PFS, with adenocarcinoma patients having a higher risk of progression compared to those with squamous cell carcinoma (*p* < 0.001). Initial nodal staging was not a statistically significant prognostic parameter for PFS in this study collective (*p* = 0.369), while the existence of initial metastases carried a higher risk of later progression (*p* < 0.001).

The use of IS IGABT and the number of interstitial needles correlated positively to the size of the hrCTV volume (rs = 0.514, *p* < 0.001). There was no statistically significant correlation between the number of needles and total dose to hrCTV (r = 0.093, *p* = 0.304).

There was no correlation between the mean dose at the historically used point A and the cumulative total dose to the hrCTV (r = 0.032, *p* = 0.725). Cox regression analysis showed no significant correlation of mean dose, max dose, or cumulative dose to point A and PFS (*p* = 0.186; *p* = 0.764; *p* = 0.235) or to OS (*p* = 0.833; *p* = 0.598; *p* = 0.784). Even though the correlation of mean dose to point A and LC was statistically significant, results would imply that a higher dose to point A increases the risk of local recurrence, which is unlikely (*p* = 0.041) and most likely due to the fact that higher doses at point A result from the hrCTV volume exceeding point A caused by extensive tumor spread, which is associated with worse LC. There was no correlation of maximum dose at point A from single brachytherapy fractions or cumulative dose from all brachytherapy fractions to point A and LC (*p* = 0.612; *p* = 0.118).

Treatment-related toxicities were grouped into gastrointestinal, lower urinary tract, and vaginal categories. Gastrointestinal toxicity (GI toxicity) included symptoms such as meteorism, diarrhea, and proctitis including defecation pain. Lower urinary tract toxicity encompassed pollakiuria, nycturia, incontinence, dysuria, and cystitis. Vaginal toxicity was defined by mucositis, pruritus, stenosis, and discharge.

Among the 146 patients evaluated for acute toxicity, 75% experienced GI toxicity, with 40% presenting mild symptoms (CTCAE °I), 25% moderate (CTCAE °II), and 9% severe (CTCAE °III). Lower urinary tract toxicity was reported in 68% of patients, with 39% experiencing mild (CTCAE °I) and 29% moderate (CTCAE °II) symptoms; no severe cases (CTCAE °III) were documented. Vaginal toxicity occurred in 50% of patients, with 23% having mild symptoms (CTCAE °I) and 27% having moderate symptoms (CTCAE °II). The prevalence of acute toxicity is depicted in Table 5.

For chronic toxicity assessed in 89 patients, at least three months after the end of treatment, GI toxicity was observed in 61% of patients, with 35% experiencing mild symptoms (CTCAE °I), 21% moderate (CTCAE °II), and 4% severe (CTCAE °III), of which two patients reported fecal urgency with stool incontinence and two patients suffered from increased stool frequency (more than 7 defecations a day). Lower urinary tract toxicity was reported in 64% of patients, with 36% mild (CTCAE °I) and 28% moderate (CTCAE °II). Vaginal toxicity was the most prevalent chronic side effect, affecting 87% of patients; 45% experienced mild symptoms (CTCAE °I), and 42% had moderate symptoms (CTCAE °II). The prevalence of chronic toxicity is depicted in Table 6.

A total of 16 patients (18%) experienced sacral insufficiency fractures. Of these, 7 patients (8%) had fractures only detectable by MRI (CTC °I), while 9 patients (10%) had symptomatic fractures (CTC °II). Chronic Lymphedema was prevalent in 39 patients (44%), 25 of these patients were staged by pelvic lymphonodectomy before treatment initiation. Polyneuropathy (PNP) was observed in 14 patients (16%), potentially related to the chemotherapy regimen. A relevant post-therapeutic worsening of the glomerular filtration rate of at least −20 mL/min resulting in a GFR < 65 mL/min was observed in 11 of 78 assessed patients (14%).

## 4. Discussion

With a large cohort of 149 patients treated at a single institution, this is one of the largest monocentric studies in the field of IGABT for LACC. The availability of original radiation plans allows for detailed dosimetric analysis and comparison, and the monocentric setting ensures consistency in treatment protocols and follow-up procedures. However, the retrospective nature of the study is a limitation, as it introduces the possibility of selection bias and incomplete data, especially to loss of follow-up. An MRI was not necessarily performed at the first brachytherapy fraction, which is a critical time for optimal hrCTV definition. This gap may have influenced the dosimetric parameters and subsequently the clinical outcomes, potentially contributing to the comparatively poor local control observed in some cases.

We included six patients with oligometastatic disease who received definitive chemoradiotherapy with curative intention. This might have influenced the oncological outcome of the cohort. Therefore, we performed statistical analyses without these patients and without the two patients with neuroendocrine tumors, who are also known to have a poorer prognosis. When excluding these patients (n = 8), the oncological outcome of the cohort was only marginally different: OS was 87%, 73%, and 66% at 1.2 and 3 years, PFS was 74%, 59%, and 49% at 1.2 and 3 years. LC was 90%, 80%, and 75% at 1, 2, and 3 years; DFS was 79%, 63%, and 57%. As we aimed to report real-world data, we included these patients in our report.

Our cohort comprises a relatively large percentage of T4 patients (15%), which is one explanation for the comparatively poor oncological outcomes. However, it has to be mentioned that only four patients were proven T4 by biopsy, while the others were diagnosed by MRI and/or cystoscopy/rectoscopy. So, the true number of T4 patients might have been lower. Correspondingly, we observed no fistula during follow-up. This has to be kept in mind when interpreting our results.

Another point to address is the high percentage of patients receiving pathological lymph node staging (63%), which caused a delay in the start of treatment and might have influenced oncological outcomes. The median time from the date of initial diagnosis until the start of radiotherapy was 39 days for patients who did not undergo LNE, whereas patients who received LNE had a median interval of 60 days. In fact, this longer time interval might be a factor influencing the oncological outcome.

The EMBRACE studies investigated the central role of brachytherapy as part of CRT in the management of LACC. EMBRACE I demonstrated that image-guided adaptive brachytherapy (IGABT) significantly improved local control and overall survival while minimizing toxicity. The five-year local control rates exceeded 90%, and 5-year overall survival rate was 74% even approaching 80% in patients with stage I–II disease. The 5-year disease-free survival was 68% [7]. Furthermore, it was shown that using IC/IS techniques and adaptive planning to enhance dose distribution can reduce treatment-related morbidity [8]. In this study, we investigated patients that were treated according to this principle. Though tumor stage was significantly more advanced in our cohort than in the EMBRACE study cohort with almost 80% of patients in FIGO stadium III and IV. Nevertheless, our study underscores the EMBRACE results in showing the effectiveness of IGABT in achieving high local control. Though the relatively high distant recurrence rates especially in patients with initial metastases (mostly paraaortal lymphnodes) showed that systemic disease control is still a critical challenge in cervical cancer management.

Other authors have shown similar results but in smaller patient collectives. For example, Rogowski et al. showed 3-year LC as well as pelvic control rates of 97.6% each and a 3-year distant metastasis-free survival of 59.9% and 3-year OS of 81.6% in 46 LACC patients treated with IGABT using the Venezia applicator [9]. Notably, in this cohort, fewer patients had advanced-stage disease compared to our collective, possibly explaining the better LC and NPFS rates; however, PFS was comparable.

Our results highlight the importance of the use of IC/IS IGABT in combination with IMRT in achieving high doses in the target volumes, thus rendering excellent oncological outcomes in LACC patients. The performed Cox regression analysis highlighted the total doses to the hrCTV from brachytherapy as a key factor influencing both LRFS and overall PFS in cervical cancer patients. A higher total dose of the hrCTV was significantly associated with a reduced risk of local recurrence and overall disease progression, highlighting the importance of adequate brachytherapy dosing in improving survival outcomes. Even though dose coverage of the target volume was improved by the use of the IC/IS technique, a larger hrCTV volume was significantly associated with an increased risk of local recurrence and a worse PFS. This indicates that patients with larger tumor volumes are at higher risk for disease progression and may require more aggressive or additional treatment strategies.

Interestingly, the initial nodal stage did not show an impact on PFS, suggesting that combined CRT treats pelvic nodal disease sufficiently. The presence of paraaortal nodal metastases or oligometastases as well as Adenocarcinoma histology significantly increased the risk of disease progression, suggesting that this patient population could profit from an intensified treatment regimen.

Regarding brachytherapy prescription, it must be noted that the use of interstitial needles leads to large variations in the dose at point A. The closer the needle is situated to point A, the higher the dosage. As shown by this study, dose to point A is not a sufficient indicator for oncological results in patients treated by IC/IS brachytherapy. Since target volume definition and target volume coverage improved substantially by the implementation of MRI-guided IGABT and IC/IS technique, point A should no longer be used for brachytherapy prescription.

After decades of sobering results from studies on improving the oncological outcome of cervical cancer patients, new approaches to enhance oncological outcomes have emerged in recent years. For example, the integration of immune checkpoint inhibitors concurrently with standard treatment has been explored in the ENGOT-cx11/GOG-3047/KEYNOTE-A18 trial. Results from two pre-planned interim analyses suggest that the use of pembrolizumab alongside CRT improves progression-free survival and overall survival for node-positive or FIGO stage III-IVA patients (according to FIGO classification from 2014), with a PFS of 68% versus 57% at 24 months [10] and an overall survival rate of 86% versus 79% at 36 months [11]. In a patient cohort with similarly advanced tumor stages, the oncological outcome of the CRT treatment group is very similar to our results. Long-term results from the Keynote-A18 trial must be awaited, but it is expected that the curves of the recurrence rates of the cohorts will diverge even further in favor of the pembrolizumab cohort. Even though immune-related adverse events were expectedly observed more frequently in the pembrolizumab group, the authors reported no significant impact on the quality of life of patients so far.

In a similar design to the KEYNOTE-A18 trial, the CALLA trial examined durvalumab in combination with CRT followed by a maintenance phase versus CRT alone [12], showing early outcomes in terms of improved PFS though not statistically significant at 24 months (65.9% vs. 62.1%). However, the integration of pembrolizumab and durvalumab must be carefully evaluated for immune-related adverse events, which could impact patient quality of life.

Another approach was investigated in the GCIG INTERLACE trial, using neoadjuvant chemotherapy (NACT) consisting of 6 courses of Carboplatin AUC2 and Paclitaxel 80 mg/m^2^ weekly followed by standard RCT (EBRT with total dosage of 40–50.4 Gy in 20–28 fractions) and BRTH with the intend to give a minimum total EQD2 dose of 78 Gy to point A [13,14]. In this cohort, most patients presented with FIGO stage IIb disease (70%), only 11% had stage IIIB disease and 43% had nodal metastases. It has to be noted, that patients with paraaortal lymph node involvement or FIGO stage IIIA disease were excluded in this trial. The results demonstrated improved 5-year-PFS rates by the use of induction chemotherapy with 72% vs. 64% (HR 0.65) as well as improved 5-year-OS rates with 80% vs. 70% (HR 0.6). For interpretation of the INTERLACE trial, it must be considered that patients were on average younger and in very good performance status with less advanced tumor stages than in our study. Only about 40% of patients were treated with IMRT, the others received EBRT using 3DCRT. Also, the brachytherapy dose technique was significantly less refined with patients receiving intracavitary brachytherapy only, mostly prescribed to point A (70%). 3D planning was recommended, but in 21% only 2D imaging was applied. 3D-IGABT with prescription to the hrCTV was applied in 30% of patients only. Median total EQD2 was 79.4 Gy (range 44.3–120.9 Gy) and for the 30% of patients treated with IGABT 87 Gy (56.3–120.9 Gy), also highlighting the benefit of IGABT in applying sufficiently high doses to the tumor. Overall, the PFS in the standard CRT cohort was notably lower than achievable regarding the cohort being mostly at low risk of systemic recurrence. Toxicity rates were relatively high in both cohorts with CTC Grade ≥ 3 adverse events in 48% of patients receiving standard therapy and 59% of patients receiving NACT. Other authors have already questioned the benefit of NACT reported in this trial when applying modern state-of-the-art radiotherapy and discussed the potential for increased overall morbidity with the integration of NACT and advanced treatment techniques [15].

Further investigations on combining NACT with CRT for the treatment of selected patients with a high risk of relapse as well as combining standard CRT with novel agents like immune-checkpoint inhibitors are necessary to reach a comprehensive approach for the management of both local and systemic disease control while keeping toxicity to a minimum. In any case, image-guided adaptive brachytherapy should be the standard of treatment in radiotherapy when exploring the benefit of additional treatment options.

The reported toxicity rates in our cohort were comparable to the EMBRACE I cohort with a relatively high prevalence of mild to moderate lower urinary tract toxicity in our cohort with 64% of patients experiencing any grade of urinary morbidity at follow-up as opposed to 69.8% [16]. We observed hardly any improvement from acute to persistent bladder toxicity. CTCAE grade 3 toxicity rates were comparable to the EMBRACE I cohort where 21.5% of patients experienced grade 3 or higher overall toxicity after 3 years, with 7% suffering from GI Toxicity ≥ grade 3 [17] opposed to 9% acute and 4% late grade 3 GI toxicity in our study. It must be considered that our results were mostly patient-reported and there was no baseline data available for the retrospectively analyzed patients; therefore, we considered a diarrhea frequency ≥ 5 stools per day as grade 2 and ≥7 stools per day (n = 2) or proctitis with stool incontinence (n = 2) as grade 3.

The considerable prevalence of chronic mild or moderate vaginal toxicity in our cohort is most likely due to the high rate of initial vaginal infiltration (52%). Due to the retrospective nature of this study, there were no baseline examinations which probably leads to over-estimation of late toxicity. The gynecological examinations at follow-up were performed by the radiation-oncologist at brachytherapy department and as part of the regular follow-up, which might be another reason for the relatively high rate of late vaginal toxicity CTC °II reported, compared to, for example, the EMBRACE collectives with 19% CTCAE grade 2 toxicity after 15 months compared to 42% in our cohort [18]. However, further investigation into vaginal dose constraints and protective strategies may be warranted, as these symptoms significantly impact the quality of life.

Our study results suggest that even though the incorporation of adaptive image-guided radiotherapy has somewhat mitigated acute toxicity, a significant burden still exists, especially in patients with locally very advanced disease (FIGO stages III and IVa).

Our study uniquely reported on sacral insufficiency fractures, chronic lymphedema, polyneuropathy (PNP), and chronic kidney injury, which were not as extensively detailed in EMBRACE or INTERLACE. Sacral insufficiency fractures were observed in 18% of patients, with 10% of patients reporting symptoms. This complication is underreported in other studies but represents a significant morbidity in our cohort, potentially related to the high cumulative radiation doses delivered to the pelvic bones. Chronic lymphedema was prevalent in 44% of patients, particularly among those who underwent pelvic lymphadenectomy before treatment, underscoring the importance of careful consideration of surgical staging especially when the application of a simultaneous integrated nodal boost is an option.

These findings also highlight the importance of comprehensive pre-treatment assessment and close monitoring for the early identification and management of toxicities. Future studies should focus on optimizing treatment regimens to balance efficacy and toxicity, incorporating patient-reported outcomes to better understand the impact of these toxicities on quality of life.

## 5. Conclusions

These results emphasize the importance of individualized treatment planning in LACC by applying interstitial IGABT for optimizing brachytherapy dosing and managing larger tumor volumes to reduce the risk of local recurrence in patients with advanced uterine cervical cancer.

The dosage points used historically are no longer relevant for modern IGABT. The size of the hrCTV, representative of the tumor volume, and the dose of the hrCTV are prognostically relevant parameters. By using combined IC/IS IGABT, adequate dose coverage of the hrCTV can be achieved even in LACC with large tumor volumes, which is crucial for LC. Regarding the burden of systemic recurrence, especially in node-positive patients, further investigations into adding new agents to the standard CRT should be conducted, taking into account the need to balance effectiveness and toxicity.

## Figures and Tables

**Table 1:** Patient demographics and tumor characteristics. Median and interquartile range (IQR) are specified where applicable; number of cases and percentages are stated.

	Median (IQR)	Cases	%
Age at diagnosis	55 (31–88)	149	
Histology			
Adenocarcinoma		23	15%
Squamous Cell Carcinoma		124	83%
Neuroendocrine Carcinoma		2	1%
Clinical Tumor Stage			
T1b		21	14%
T2a		18	12%
T2b		68	46%
T3a		2	1%
T3b		18	12%
T4		22	15%
Lymphnode Stage			
N0		43	29%
N1		106	71%
Metastasis			
M0		107	72%
M1		42	28%
M1a *		39	26%
M1b *		1	1%
M1c *		5	3%
Grading		135	
G1		5	4%
G2		70	52%
G3		60	44%
Staging by Lymphonodectomy			
Pelvic		79	53%
Paraaortal		75	50%
Organ infiltration			
Vagina		78	52%
Bladder		18	12%
Rectum		6	4%

* The site of each distant metastasis is displayed; there were patients who had paraaortal lymph node metastases and simultaneous other distant metastases.

**Table 2:** Median and interquartile range (IQR) for dose–volume parameters for high-risk CTV (hrCTV), planning target volume (PTV), as well as organs at risk (bladder, rectum, sigma, bowel). The third column states the number of cases for which the information was available. The table is divided into the following sections: for single brachytherapy fraction, the cumulative dose from brachytherapy, dose–volume parameters from external beam radiotherapy (EBRT), and total dose from brachytherapy and EBRT.

	Median (IQR)	Cases
Dose–Volume Parameters per Brachytherapy Fraction		
hrCTV		146
Volumen (cm^3^)	33.46 (24.32–50.4)	
D98 (Gy EQD2_10_)	8.72 (7.67–9.73)	
D90 (Gy EQD2_10_)	10.78 (9.86–11.66)	
Bladder		147
D0.1cc (Gy EQD2_3_)	13.28 (11.98–14.04)	
D1cc (Gy EQD2_3_)	10.94 (9.93–11.69)	
D2cc (Gy EQD2_3_)	9.96 (9.00–10.75)	
Rectum		147
D0.1cc (Gy EQD2_3_)	7.73 (6.24–8.92)	
D1cc (Gy EQD2_3_)	5.79 (4.78–6.98)	
D2cc (Gy EQD2_3_)	5.06 (4.17–6.04)	
Sigma		147
D0.1cc (Gy EQD2_3_)	7.55 (5.26–9.08)	
D1cc (Gy EQD2_3_)	5.51 (4.02–6.74)	
D2cc (Gy EQD2_3_)	4.75 (3.38–5.68)	
Bowel		141
D0.1cc (Gy EQD2_3_)	5.68 (2.74–7.62)	
D1cc (Gy EQD2_3_)	4.27 (2.02–5.61)	
D2cc (Gy EQD2_3_)	3.43 (1.79–4.97)	
Point A		147
Mean (left/right) (Gy EQD2_10_)	8.25 (5.67–9.58)	
Cumulative Dose–Volume Parameters from Brachytherapy		
hrCTV D90 (Gy EQD2_10_)	43.44 (40.96–45.9)	124
Bladder D2cc (Gy EQD2_3_)	39.44 (24.15–66.52)	139
Rectum D2cc (Gy EQD2_3_)	20.13 (9.46–40.05)	139
Sigma D2cc (Gy EQD2_3_)	18.84 (3.82–36.53)	138
Bowel D2cc (Gy EQD2_3_)	15.64 (2.85–32.39)	102
Point A		142
Mean (left/right) (Gy EQD2_10_)	46.32 (29.25–65.81)	
Dose–Volume Parameters from EBRT		
PTV		133
Volume (cm^3^)	1695 (546–4485)	
D50 (Gy EQD2_10_)	44.25 (44.25–47.13)	
Bladder		132
Volume (cm^3^)	254 (48–1107)	
D50 (Gy EQD2_3_)	39.27 (23.93–82.59)	
Rectum		131
Volume (cm^3^)	72 (25–283)	
D50 (Gy EQD2_3_)	42.12 (3.04–73.1)	
Sigma		57
Volume (cm^3^)	91 (15–322)	
D50 (Gy EQD2_3_)	42.22 (22.92–48.67)	
Bowel		132
Volume (cm^3^)	1314 (11–3994)	
D50 (Gy EQD2_3_)	19.91 (3.81–48.36)	
Total Dose from EBRT and Brachytherapy		113
hrCTV (Gy EQD2_10_)	88.5 (85.75–91.92)	113
≥85 Gy EQD2_10_		97 (86%)
<85 Gy EQD2_10_		16 (14%)
Bladder (Gy EQD2_3_)	77.46 (55.26–96.74)	126
Rectum (Gy EQD2_3_)	61.00 (34.93–82.06)	125
Sigma (Gy EQD2_3_)	59.68 (27.65–74.71)	53
Bowel (Gy EQD2_3_)	34.70 (15.16–58.79)	93

Abbreviations: hrCTV = high-risk clinical target volume; PTV = planning target volume; D2cc, D1cc, D0.1cc = dose received by the most irradiated 2 cubic centimeters (cc), 1 cc, and 0.1 cc of a specific organ; EQD2_3_ = equal dose in 2 Gy fractions with α/β of 3 for normal tissue; EQD2_10_ = equal dose in 2 Gy fractions with α/β of 10 for tumor.

**Table 3:** Cox–Proportional–Hazard Model evaluating the influence of total biologically equivalent dose from brachytherapy (BRTH) and external beam radiotherapy (EBRT) to the high-risk clinical target volume (hrCTV), the volume of hrCTV, tumor histology (Squamous cell carcinoma or Adenocarcinoma), and concurrent application of chemotherapy on local control.

Variable	Regression Coefficient	Hazard Ratio	95% Confidence Interval	*p*-Value
Total dose from BRTH and EBRT to hrCTV	−0.186	0.830	0.734–0.939	0.003
hrCTV volume	0.032	1.032	1.010–1.056	0.005
Histology type	0.201	1.223	0.305–4.897	0.777
Concurrent chemotherapy	−2.102	0.122	0.014–1.059	0.122

**Table 4:** Cox–Proportional–Hazard Model evaluating the influence of total biologically equivalent dose from brachytherapy (BRTH) and external beam radiotherapy (EBRT) to the high-risk clinical target volume (hrCTV), the volume of hrCTV, concurrent application of chemotherapy, tumor histology (Squamous cell carcinoma or Adenocarcinoma), nodal stage, and initial presence metastasis on progression-free survival.

Variable	Regression Coefficient	Hazard Ratio	95% Confidence Interval	*p*-Value
Total dose from BRTH and EBRT to hrCTV	−0.085	0.919	0.859–0.982	0.013
hrCTV volume	0.019	1.019	1.003–1.035	0.020
Histology type	1.204	3.333	1.737–6.397	<0.001
Concurrent chemotherapy	−0.376	0.687	0.058–8.165	0.766
Nodal stage	0.486	1.626	0.564–4.689	0.369
Metastasis stage	1.221	3.392	1.665–6.910	<0.001

**Table 5:** Acute toxicity (up to 6 weeks after end of treatment).

n = 146	Gastrointestinal Toxicity		Lower Urinary Tract Toxicity		Vaginal Toxicity	
Any grade	109	75%	99	68%	74	50%
CTCAE °I	59	40%	57	39%	34	23%
CTCAE °II	37	25%	42	29%	40	27%
CTCAE °III	13	9%	0	0%	0	0%

Abbreviation: CTCAE = Common Terminology Criteria for Adverse Events classification (v5).

**Table 6:** Chronic toxicity (>3 months after end of treatment).

n = 89	Gastrointestinal Toxicity		Lower Urinary Tract Toxicity		Vaginal Toxicity	
Any grade	54	61%	57	64%	77	87%
CTCAE °I	31	35%	32	36%	40	45%
CTCAE °II	19	21%	25	28%	37	42%
CTCAE °III	4	4%	0	0%	0	0%

Abbreviation: CTCAE = Common Terminology Criteria for Adverse Events classification (v5).

## Data Availability

The datasets presented in this article are not readily available because of limitations due to national data protection requirements. Requests to access the datasets can be directed to the corresponding author.

## Abbreviations

The following abbreviations are used in this manuscript:

CRT	Radiochemotherapy
DPFS	Distant progression-free survival
EBRT	External beam radiotherapy
ESGO	European Society of Gynecological Oncology
ESTRO	European Society of Radiotherapy and Oncology
EQD2	Equivalent dose in 2 Gy fractions
FIGO	The International Federation of Gynecology and Obstetrics
GEC ESTRO	Groupe Européen de Curiethérapie and the European Society for Radiotherapy and Oncology
GI toxicity	Gastrointestinal toxicity
hrCTV	High-risk clinical target volume
IC/IS	intracavitary and interstitial
IGABT	Image-guided adaptive brachytherapy
LACC	Locally advanced cervical cancer
LC	Local control
NACT	Neoadjuvant chemotherapy
NPFS	Nodal progression free survival
OAR	Organs at risk
OS	Overall survival
PFS	Progression-free survival
PTV	Planning target volume
VMAT	Volumetric arc therapy
